# One-step large-scale deposition of salt-free DNA origami nanostructures

**DOI:** 10.1038/srep15634

**Published:** 2015-10-23

**Authors:** Veikko Linko, Boxuan Shen, Kosti Tapio, J. Jussi Toppari, Mauri A. Kostiainen, Sampo Tuukkanen

**Affiliations:** 1 Aalto University, Department of Biotechnology and Chemical Technology, Biohybrid Materials, Espoo, P.O. Box 16100, FI-00076 Aalto, Finland; 2 University of Jyvaskyla, Department of Physics, Nanoscience Center, Jyväskylä, P.O. Box 35, FI-40014 University of Jyväskylä, Finland; 3 Tampere University of Technology, Department of Automation Science and Engineering, Tampere, P.O. Box 692, FI-33101, Finland

## Abstract

DNA origami nanostructures have tremendous potential to serve as versatile platforms in self-assembly -based nanofabrication and in highly parallel nanoscale patterning. However, uniform deposition and reliable anchoring of DNA nanostructures often requires specific conditions, such as pre-treatment of the chosen substrate or a fine-tuned salt concentration for the deposition buffer. In addition, currently available deposition techniques are suitable merely for small scales. In this article, we exploit a spray-coating technique in order to resolve the aforementioned issues in the deposition of different 2D and 3D DNA origami nanostructures. We show that purified DNA origamis can be controllably deposited on silicon and glass substrates by the proposed method. The results are verified using either atomic force microscopy or fluorescence microscopy depending on the shape of the DNA origami. DNA origamis are successfully deposited onto untreated substrates with surface coverage of about 4 objects/mm^2^. Further, the DNA nanostructures maintain their shape even if the salt residues are removed from the DNA origami fabrication buffer after the folding procedure. We believe that the presented one-step spray-coating method will find use in various fields of material sciences, especially in the development of DNA biochips and in the fabrication of metamaterials and plasmonic devices through DNA metallisation.

DNA has astonishing potential as programmable nanoscale construction material for the bottom-up-based nanofabrication^1^ and for a great variety of bionanotechnological applications^2^. To date, a plethora of design strategies for assembling DNA molecules into customized structures and templates have been introduced^2^. Arguably, one of the most elegant one is a scaffolded DNA origami technique, which enables a straightforward fabrication of arbitrary two- (2D)^3^ and three-dimensional (3D)^4^,^5^ nanoshapes, meshed structures^6^ as well as large assemblies with high spatial addressability^7^.

Recently, DNA nanoarchitectures have been utilized in various innovative applications that truly underline the feasibility of the structural DNA nanotechnology. DNA origamis can serve as molecular scale circuit boards in nanoelectronics^8^, scaffolds for plasmonic structures^9^,^10^ and gatekeepers for solid-state nanopores^11^,^12^. Biotechnological examples include smart molecular devices^13^,^14^ such as nanorobots^15^, cellular delivery vehicles^16^,^17^, and synthetic ion channels^18^.

Despite the fact that DNA origami itself has limited properties in optics and in electronics^19^,^20^,^21^,^22^, its use in templating is extremely promising. There exist plenty of placement and deposition methods for DNA origamis that are useful for patterning on different substrates. One straightforward way is to utilize chemically modified surfaces, which enable selective anchoring of the origamis^23^,^24^,^25^, whereas some substrates can be used to assist large-scale lattice formation of DNA origamis^26^,^27^,^28^. In addition, hierarchically ordered nanosystems can be obtained by combining DNA structures with patterned substrates, such as lithographically fabricated confined wells^29^,^30^ and other surface patterns, which are specifically designed for the alignment of DNA structures^31^,^32^. Moreover, single DNA molecules^33^,^34^,^35^ and complex DNA nanoarchitectures^36^,^37^,^38^ can be selectively guided and anchored onto substrates by means of electric fields. Nevertheless, in order to utilize such approaches one has to pay extra attention to the deposition conditions (salt concentration, pH, surface chemistry etc.). In many occasions, the required treatments and prevalent conditions set strict limitations to the conceivable applications.

In general, print-coating technologies provide high-throughput and low-cost patterning methods for solution processable materials^39^. Although aqueous dispersion of DNA is solution processable, these techniques have not been so far adapted in the field of structural DNA nanotechnology. However, in this article we show that the high spatial addressability of the structurally different DNA origamis can be genuinely combined with the large-scale print-coating methods. The authors have previously demonstrated the feasibility of the solution processing techniques in various other applications, such as stretchable electrodes^40^, piezoelectric sensors^41^,^42^, transparent touch panels^43^, supercapacitors^44^,^45^ and energy harvesters^46^,^47^. The same methods are now expanded to the field of DNA nanotechnology.

In this study a spray-coating technique is used to achieve a straightforward, fast and cost-effective way to pattern substrates with custom-shaped DNA structures on large scale (Fig. 1). The deposition areas demonstrated here are ~10 cm^2^, but the method can be easily scaled-up to wafer-scales and even larger surfaces. The proposed method does not require special substrate conditions or washing procedures, and moreover, salt residues from the DNA origami buffer can be eliminated by removing salt ions right before the coating procedure. With this method, the fabricated DNA origami dispersions can be utilised fully without any loss of material, which makes the relative cost of DNA reagents negligible. Thus, this highly robust technique could find use in many applications. Homogeneous large-scale DNA patterns could be exploited e.g. in creating DNA origami microarrays^48^, whereas DNA origami-based metallic assemblies^49^ can yield intriguing opportunities for developing nanoplasmonics^10^,^50^ and metamaterials^51^.

## Results

### DNA origami nanostructures

**Figure 1:**
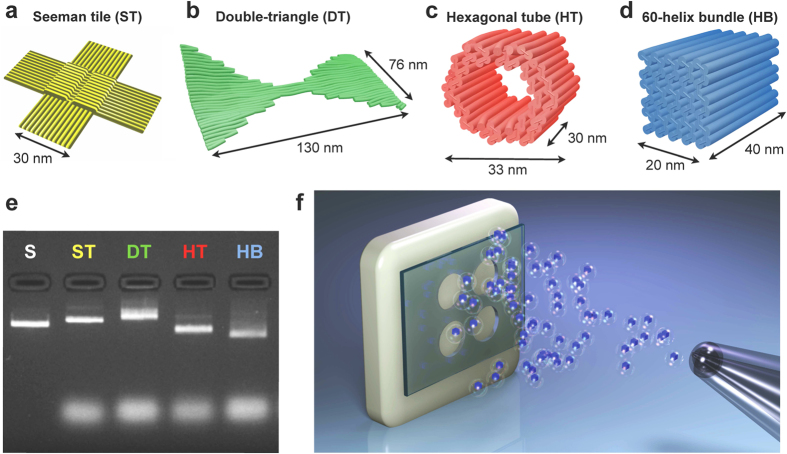
DNA origami nanostructures for patterning. (**a**) Seeman tile (ST)^7^, (**b**) double-triangle (DT), (**c**) hexagonal tube (HT)^52^ and (**d**) 60-helix bundle (HB). (**b**–**d**) are CanDo-simulated^57^ deformed solution shapes, resolved using caDNAno design files^58^ as inputs. (**e**) Agarose gel electrophoresis of the well-folded DNA structures described in (**a**–**d**). S = scaffold strand (M13mp18), which is used as a reference sample. (**f**) A schematic view of patterning a substrate (white colour) through an optionally used mechanical mask (green colour, openings 1–2 mm in diameter). DNA origami nanostructures (blue 60-helix bundles) are sprayed onto the substrate using an airbrush device. Figure (**f**) is not in scale.

In this work, four different DNA origami structures were prepared by folding the M13mp18 scaffold strand with the help of specific sets of staple strands. 2D single-layer DNA architectures, a Seeman tile (ST)^7^ and a double-triangle (DT) were fabricated for studying the coating with scanning probe microscopy. Furthermore, two 3D multilayer and fluorescently labelled DNA architectures, a hexagonal tube (HT)^52^ and a 60-helix bundle (HB) were utilised when the coating was characterised using optical microscopy. The DNA origami shapes and their dimensions are presented in Fig. 1a–d. The correct folding of all four DNA objects was verified using agarose gel elecrophoresis (Fig. 1e) by comparing the running speed of the leading bands to the reference sample (M13mp18 scaffold). In addition, correct HT and HB folding was confirmed using transmission electron microscopy (Supplementary Fig. S1 and Supplementary Note 2).

**Figure 2:**
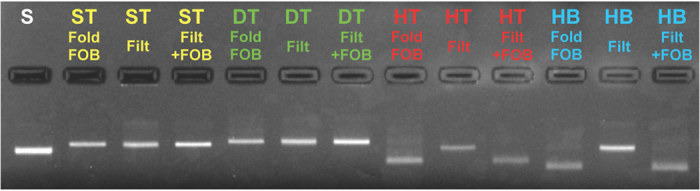
Agarose gel electrophoresis for all four types of DNA origami structures. Fold = DNA structures after folding in the folding buffer (FOB) (for ST and DT FOB is 1× TAE + 12.5 mM Mg^2+^ and for HT and HB 1× TAE + 20 mM Mg^2+^). Filt = DNA structures filtered with pure water. Filt+FOB = Filtered DNA structures stored in water for 1 day, after which the buffer conditions have been adjusted to the same as for folding. S is a scaffold strand (M13mp18), which is used as a reference sample.

The excess DNA staple strands (bright areas at the bottom of the gel lanes (Fig. 1e)) were removed in a nondestructive spin-filtering process. In general, spin-filtering technique is a straightforward way for exchanging the buffer and for efficiently removing salt from the starting solution^36^. For filtering of the samples, pure water was used, which practically removes all the Mg^2+^ ions and other salt residues from the DNA origami annealing solution (see Methods section for the details). Mg^2+^ concentration changes from 12.5–20 mM to as low as 1–4 *μ*M. Importantly, agarose gel electrophoresis analysis (Fig. 2) proves that the DNA structures stay intact in the purification process. Although HT and HB bands shift in the gel after water-filtering (due to the different buffer conditions), the bands appear again at the exactly same positions once the salt and buffer have been added, i.e. the folding conditions have been restored.

The agarose gel analysis was repeated 14 weeks later for the spin-filtered samples in water (Supplementary Fig. S2). It showed that the DNA origamis are stable in water over long time. However, it is probable that slight agglomeration of the objects starts to occur over time if the storage solution has a high origami concentration. Hence, all the spray-coating experiments reported in this article were performed immediately after the purification step.

### Spray-coating and patterning with DNA nanostructures

The purified and salt-free DNA origamis (in water) were deposited using a spray-coating method (Fig. 1f). The spray-coating was performed using a manual airbrush which was kept at 5–10 cm distance from the substrate. The coating was carried out in a layer-like fashion by spraying a small layer at a time. The droplets were let to dry between the successively deposited layers. In this work, two different approaches were applied; first, we aimed at homogeneous coverage of 2D ST and DT origamis on silicon substrates (without masking), and second, formation of large-area patterns of fluorescently labelled 3D HT and HB origamis (with masking).

### Homogeneous substrate coverage using DNA origamis

**Figure 3:**
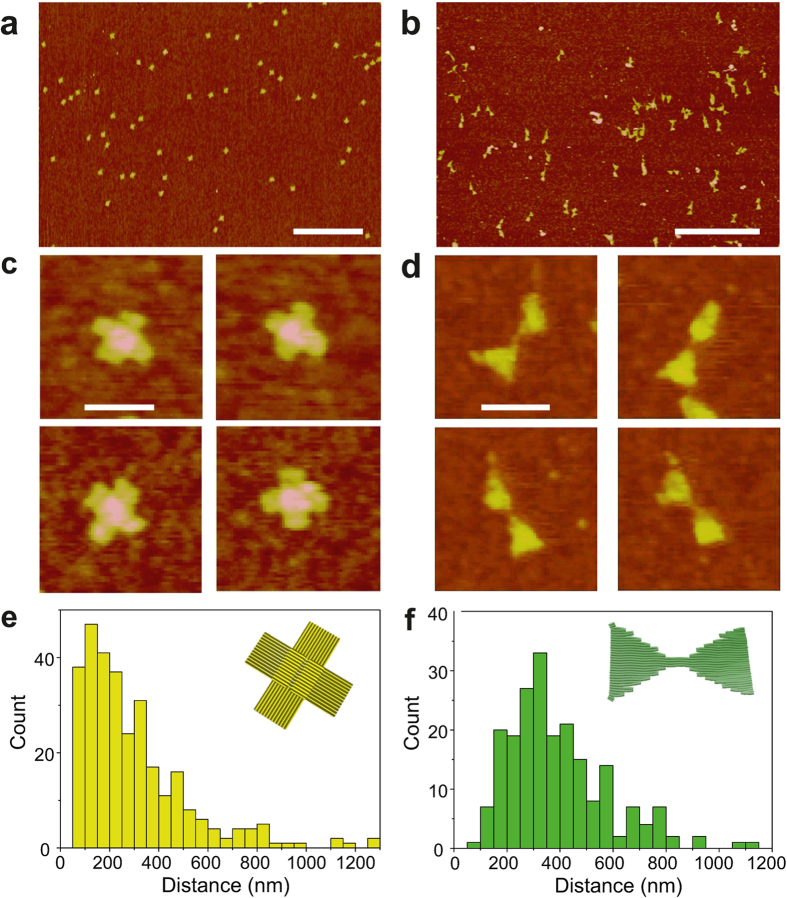
Purified and salt-free 2D DNA origami nanostructures deposited onto the untreated silicon substrates using a spray-coating technique. AFM images of (**a**) Seeman tile (ST) and (**b**) double-triangle (DT) coated substrates. The scale bars are 1 *μ*m. (**c**,**d**) Zoomed-in images of single objects. The scale bars are 100 nm. (**e**,**f**) Histograms of the nearest-neighbour distances for deposited ST and DT origami shapes. The deposition was carried out using optimised parameters for homogeneous coating.

The two single-layer DNA origamis (ST and DT) were deposited on untreated silicon substrates by spray-coating the dilute DNA origami solutions (1 nM). The homogeneous coverage was obtained for both ST and DT origamis as can be seen from the atomic force microscope (AFM) images (Fig. 3a–d) and the histograms showing the nearest-neighbour distances (Fig. 3e,f) obtained with the coating parameters optimised for the homogeneous coverage (see the Methods section for the details). In addition, spray-coating with the optimised parameters resulted in the surface coverage values of 4.6 ± 0.8 objects/*μ*m^2^ (standard deviation, s.d.) and 3.9 ± 0.4 objects/*μ*m^2^ (s.d.) for ST and DT, respectively. The statistics of the particle coverage distribution and the nearest-neighbour distances were determined from several different AFM images.

Further, since the structures used in these experiments were flat, the structural details of the DNA objects could be fully resolved by AFM. For both structures the correct DNA origami shapes are well preserved as seen in Fig. 3c,d. This is a significant observation, taking into account that the salt has been removed from the deposition solution. The DT structure is highly flexible (the most flexible among these four structures, see Supplementary Fig. S3), and thus some objects tend to adopt slightly bent conformations on the substrate. In addition, some DT bundles were observed, but these small aggregates are formed already in the folding process via unspecific base stacking interactions between the objects (this can be seen as a faint multimer-tail in the DT lane in Fig. 1e).

### Large-area patterning with DNA origamis

**Figure 4:**
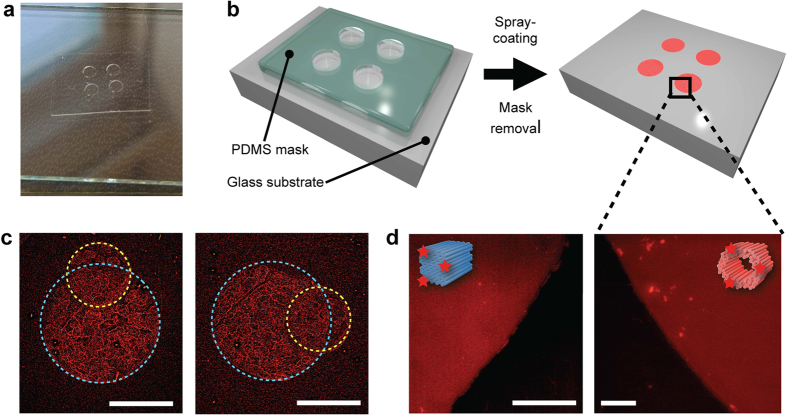
Patterning substrates with fluorescently labelled DNA origamis. (**a**) A photograph of a mechanical polydimethylsiloxane (PDMS) mask (with round 1.8 mm diameter openings) on top of the glass substrate. (**b**) A schematic view of the patterning setup. Spray-coating of labelled DNA origamis is carried out through the PDMS mask. (**c**) Fluorescence micrographs of patterns created using sequential spray-coating of Cy5-labelled and purified 60-helix bundles (HB) onto a glass substrate. HBs were deposited first through a mask with large holes (1.8 mm in diameter, blue dashed circle) and after that through a mask having slightly smaller holes (1 mm in diameter, yellow dashed circle). The scale bars are 1 mm. (**d**) Fluorescence micrographs of the glass substrates after coating with Cy5-labelled and purified HBs (left) and hexagonal tubes (right). Micrographs in (**d**) were taken after the mask removal at the edges of the formed round patterns (diameter 1.8 mm) as indicated by the black square and the dashed lines. The scale bars are 50 *μ*m.

The patterns of two fluorescently labelled (Cy5) 3D multilayer DNA origamis (HT and HB) were deposited onto glass slides through a mechanical polydimethylsiloxane (PDMS) mask (Fig. 4a,b). HT and HB structures were deposited at 10 nM concentration (10× more concentrated than the solutions used in coating of silicon substrates) in order to get enough contrast in fluorescence imaging. After coating and mask removal, the formation of HT and HB patterns on transparent glass substrates were verified using optical fluorescence microscopy. The characterisation shows that the PDMS mask edges are very sharp and the fluorescence is fairly distributed in the patterned area (Fig. 4c,d). In Fig. 4c 60-helix bundles with different concentrations (first 10 nM and second 5 nM) are deposited sequentially onto the glass substrate through two different masks (first through a mask with larger holes and then through a mask with smaller holes). Optical microscopy was used here instead of AFM in order to demonstrate the feasibility of the method also on sub-millimeter and millimeter scale. Moreover, the structural details of 3D DNA origamis are hardly visible in AFM.

The PDMS mask attaches well to flat substrates, such as silicon, glass or plastic, preventing the spray-coated drops from penetrating between the mask and the substrate^45^. In addition, the removal of the mask does not harm the obtained DNA origami pattern, and the same mask can be used several times for coating. The pattern design can be arbitrarily chosen, and the silicon mold for the mask can be prepared with high accuracy by exploiting conventional microfabrication methods. In addition, as shown in Fig. 4c, the patterning can be realised in a sequential fashion, enabling the controllable formation of complex patterns. These results show that the proposed method is highly applicable for a large-scale substrate patterning with DNA origami nanostructures.

## Conclusions

As a conclusion, we have shown that structurally distinct DNA origami nanostructures can be uniformly deposited on different substrates. The proposed deposition method is versatile and has outstandingly high yield, since all the material placed in the spray-coating device will be homogeneously deposited onto the selected substrates, opposite to the common drop casting techniques. This is also a straightforward coating method, since it does not require additional washing steps or optional pre-treatments of the substrates. Furthermore, the method enables wafer-scale deposition and patterning of DNA origamis within minutes making the method highly cost-effective. This is an important detail in the field of structural DNA nanotechnology, since large-scale fabrication of complex DNA nanostructures is still relatively expensive^53^. Thus, the efficient large-scale deposition methods are urgently needed for conceivable applications. An estimated cost of large-scale DNA origami coating with the surface coverage obtained in this work (about 4 objects/*μ*m^2^) would be of the order of 1 euro per square meter, which is rather inexpensive compared to typical substrate costs.

The presented method can be easily scaled up, and interestingly, it also enables the utilisation of flexible substrates, such as plastics, making the technique truly versatile. This feature could be exploited in a roll-to-roll production line, where large surfaces can be coated with homogeneous films. The method has potential implementations for example in creating large-scale DNA chips and self-assembly -based biosensors on specific substrates^54^. Further, metallisation of DNA nanoshapes^49^,^55^ might enable fabrication of large films with attractive plasmonic or electrical properties.

## Methods

### DNA origami preparation

DNA origami nanostructures (Fig. 1a–d) were fabricated, analysed and purified as described below.

### DNA origami folding

A double-triangle structure (Supplementary Fig. S3 and S4) was folded in 100 *μ*l quantities using 20 nM M13mp18 scaffold strand (New England Biolabs or Tilibit Nanosystems) and a set of 163 staple strands (Supplementary Table S3) (IDT, standard desalting) at 10× excess (200 nM). The design contains skipped scaffold bases to compensate the undesired global twisting of the object (the twist is caused by the square packaging of helices^56^) (Supplementary Fig. S4). The folding took place in a buffer containing 1× TAE (40 mM tris(hydroxymethyl)aminomethane (Tris), 1 mM ethylenediaminetetraacetic acid (EDTA), and acetic acid for adjusting pH to 8.3), 5 mM NaCl and 12.5 mM MgCl_2_. The thermal folding ramp (for G-Storm G1 thermal cycler) is shown below:

- From 90 °C to 70 °C: 0.2 °C decrease/8 seconds.
- From 70 °C to 60 °C: 0.1 °C decrease/8 seconds.
- From 60 °C to 27 °C: 0.1 °C decrease/2 minutes.
- Store at 12 °C.

For a 60-helix bundle (Supplementary Fig. S5), 141 staple strands (Supplementary Table S4) and additional 6 linker strands (Supplementary Table S2 and Supplementary Note 4) for anchoring Cy5-labelled strands (Supplementary Note 4) were used (IDT, standard desalting). The reagents and quantities are the same as above, except that the folding buffer contained 20 mM MgCl_2_. The folding steps are listed below:

- From 65 °C to 59 °C: 1.0 °C decrease in 15 minutes.
- From 59 °C to 40 °C: 0.25 °C decrease in 45 minutes.
- Store at 12 °C.

A Seeman tile^7^ and a hexagonal tube^52^ were folded similarly as reported in the original articles. Sidestrands for both structures were omitted in order to avoid blunt-end stacking of the objects. For HT, 5 linker strands (Supplementary Table S1 and Supplementary Note 4) were used for anchoring the Cy5-labelled strands (Supplementary Note 4).

### Agarose gel electrophoresis

Agarose gel electrophoresis (Fig. 1e) was used for verifying the quality of the folding. 2 grams of agarose (Sigma-Aldrich) was mixed with 100 ml of 1× TAE buffer containing 11 mM MgCl_2_, and the gel was stained with 80 *μ*l of ethidium bromide (EthBr) solution (0.625 mg/l). The samples were prepared by mixing 10–20 *μ*l of concentration-adjusted DNA origami solution with 2–4 *μ*l of 6× Blue Loading Dye (New England Biolabs). 10 *μ*l of each sample was loaded into a gel pocket. M13mp18 scaffold strand was used as a reference. 1× TAE including 11 mM MgCl_2_ was used as a running buffer and the gel was run with a constant voltage of 90–95 V for 45–70 minutes.

### DNA origami purification

After verifying the quality of folding, the excess amount of staple strands and unbound Cy5-labeled strands as well as salt residues were removed from the DNA origami solution in a non-destructive spin-filtering process. For filtering, we used 0.5 ml filter columns with 100 kDa molecular weight cut-off (Millipore Amicon Ultra YM-100). Filtration steps (3 filtering rounds) are described below.

- 50 *μ*l of DNA solution was diluted to 500 *μ*l in a filter column placed in a 2 ml tube using Milli-Q water, and the diluted solution was spun with 14,000 rcf for 3 minutes.
- A flowthrough was discarded and 450 *μ*l of water was added to the filter.
- Sample was spun another time repeating the steps described above. For the third similar filtering round the centrifugation time was adjusted to 5 minutes.
- Finally, the filter was turned upside down in a fresh 2 ml tube and was spun 2 minutes at 1,000 rcf in order to collect the solution from the filter membrane.

After each filtration round the volume of the solution was brought down to 15–20 *μ*l. Therefore, it can be estimated that by 3 filtering rounds the Mg^2+^ concentration can be reduced from 12.5–20 mM to 1–4 *μ*M. The concentration of the final DNA origami solution was estimated to be around 20 nM. The spray-coating was performed immediately after the purification step in order to prevent possible aggregation of the DNA objects (Supplementary Fig. S2 and Supplementary Note 3)

### Spray-coating of DNA origamis

DNA origami nanostructures were spray-coated on silicon and glass substrates using two different approaches as described below.

### Films of single-layer DNA origamis

Spray-coating of the DNA origami solutions on silicon substrate (chips of size 10 mm × 10 mm, total spraying area roughly 30 mm × 30 mm) was carried out with a manual airbrush (nozzle size 300 *μ*m) using compressed air (pressure ~3 bar) as a carrier gas. The coating was performed sequentially by depositing a layer of droplets (barely visible to eye) at the time. After every deposition layer, droplets were let to evaporate in ambient conditions. Typically the substrates were fully patterned within several minutes. The films of Seeman tile (ST) and double-triangle (DT) were deposited on silicon substrates (without masking) using 40 *μ*l of 1 nM DNA origami solution.

### Patterns of multilayer DNA origamis

The patterns of fluorescently labelled hexagonal tubes (HT) and 60-helix bundles (HB) were formed on glass substrates (microscope slides of size 75 mm by 25 mm). Same spray-coating conditions were used here as explained above. For each glass slide 40 *μ*l of 10 nM DNA origami solution was used. The pattern was defined by spray-coating through a 160 *μ*m thick polydimethylsiloxane (PDMS) film with round openings.

The PDMS film (Fig. 4a) was prepared from a silicone elastomer kit (Sylgard 184) using base/curing agent mix ratio 10:1. The mixture was spin-coated at 700 rpm for 1 min on a glass plate and cured 4 h at 60 °C in the oven. Round openings of 1.0 and 1.8 mm diameter were then patterned to the PDMS film using mechanical cutting tools.

### Sample characterisation

The substrates with homogeneously covered single-layer DNA origami structures (ST and DT) were characterised using atomic force microscopy (AFM). For multilayer DNA structures (HT and HB), fluorescence microscopy was utilized. The structural characterisation of multilayer origamis was carried out using transmission electron microscopy (TEM) (Supplementary Fig. S1 and Supplementary Note 2).

### AFM imaging

Silicon and glass substrates with immobilized DNA origamis (ST and DT) were imaged using AFM (Veeco Dimension 3100 or 5000) in tapping mode with a scan rate of 1.0–1.5 Hz.

### Fluorescence microscopy

Glass slides patterned with fluorescently labelled DNA origamis (HT and HB) were imaged using a Nikon Eclipse Ti microscope equipped with a 4× magnification object or a BioTek Cytation 3 microscope with a 20× magnification object. Cy5 was excited using either 638  nm laser (Nikon setup) or 590 nm LED cube (BioTek setup).

## Additional Information

**How to cite this article**: Linko, V. *et al.* One-step large-scale deposition of salt-free DNA origami nanostructures. *Sci. Rep.* **5**, 15634; doi: 10.1038/srep15634 (2015).

## Supplementary Material

Supplementary Information
