# Supplementary material for: One-step large-scale deposition of salt-free DNA origami nanostructures
Source: Sci Rep. 2015 Oct 23;5:15634. doi: 10.1038/srep15634 (PMC4616047; doi:10.1038/srep15634)
Supplement: Supplementary Information [file srep15634-s1.pdf]

Supplementary Information

One-step large-scale deposition of salt-free DNA origami nanostructures

Veikko Linko[1], Boxuan Shen[2], Kosti Tapio[2], J. Jussi Toppari[2], Mauri A. Kostiainen[1]* and Sampo Tuukkanen[3]*

1 Biohybrid Materials, Department of Biotechnology and Chemical Technology, Aalto University, P.O. Box 16100, FI-00076 Aalto, Espoo, Finland

2 University of Jyvaskyla, Department of Physics, Nanoscience Center, P.O. Box 35, FI-40014 University of Jyväskylä, Finland

3 Tampere University of Technology, Department of Automation Science and Engineering, P.O. Box 692, FI-33101, Tampere, Finland

*mauri.kostiainen@aalto.fi

*sampo.tuukkanen@tut.fi

Contents

Note 1. Materials 2

Note 2. TEM imaging 2

Note 3. Stability of DNA origami nanostructures in water 3

Note 4. Details of DNA origami nanostructures 3

Supplementary information references 12

Note 1. Materials

All reagents are commercially available and applied without any further purification. In all procedures the water used was Milli-Q purified or double-distilled (ddH_2O).

Note 2. TEM imaging

Structural properties of multilayer DNA origamis were characterized with transmission electron microscopy (TEM) (see Figure S1). Micrographs were taken with Tecnai 12 Bio Twin instrument. Samples were prepared on Formvar carbon coated /carbon only copper grids (Electron Microscopy Sciences) by pipetting a 3 µl drop of the sample solution onto the grid. The droplet was left on the grid for 1 min, and after that the excess solution was blotted away using filter paper. Samples were negatively stained by first applying 3 µl of staining solution (0.5 % uranyl acetate in Milli-Q water) and then removing the excess stain again with a piece of filter paper. Additional 3 µl droplet of uranyl acetate was then pipetted onto the grid, and excess liquid was blotted away after 20 s. The samples were dried at room temperature for at least 10 min before imaging.

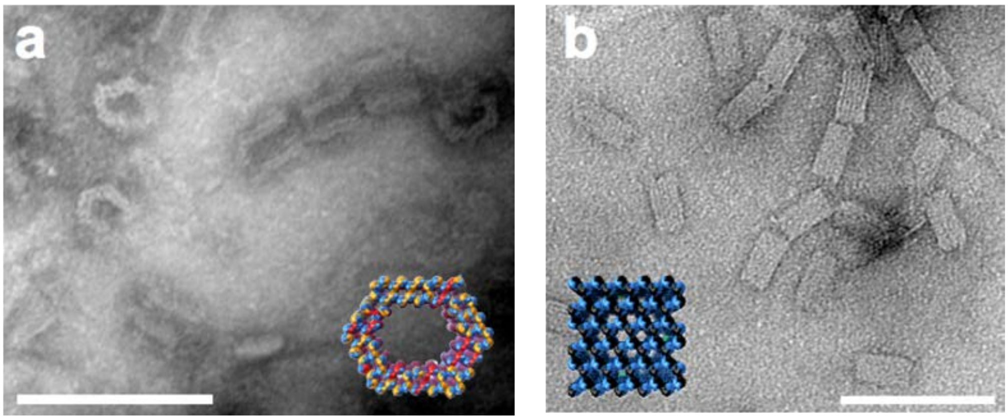

Figure S1. Transmission electron micrographs of multilayer DNA origamis used for coating experiments: **a** hexagonal tubes and **b** 60-helix bundles. The insets show the atomic models of the structures. The scale bars are 100 nm.

Note 3. Stability of DNA origami nanostructures in water

DNA origami nanostructures used in spray-coating were spin-filtered using Milli-Q water. However, this might not be an ideal buffer for storing DNA origamis over long time, since the structures might start to form aggregates. However, using low concentrations as in Fig. S2, the gel data shows that the structures can be stable even after 14 weeks of storing. Nevertheless, all the experiments reported in this article were performed right after spin-filtering.

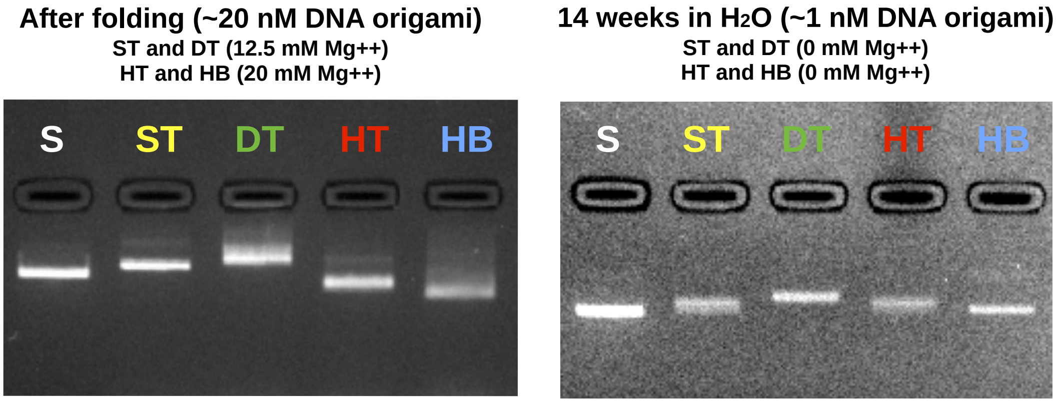

Figure S2. Agarose gel electrophoresis of the DNA origami nanostructures. The structures can stay intact in water over long time, and the agglomeration can be avoided if the low concentrations are used. Note that the structures (especially HT and HB) run with different speeds in water and in the folding buffer as explained in the main article and in Figure 2.

Note 4. Details of DNA origami nanostructures

Sequence maps and sequences for a double-triangle and 60-helix bundle are listed below. These details for a Seeman tile and a hexagonal tube have been previously published (see Supplementary Information of the publications [S1] and [S2]).

For fluorescently labeled origamis (hexagonal tube and 60-helix bundle) linker strands were used. All linkers contain a specific part that binds to the scaffold strand, and an overhanging sequence **TTTTTTCTCCTTTCCC** which binds to a Cy5-labeled fluorescent strand 5'-Cy5-GGGAAAGGAGAAAAA-3'.

Table S1. 5 linkers for a hexagonal tube.

CAACTAATCATAACCAGACGACTGGATAGCGT**TTTTTTCTCCTTTCCC**
TGGTCAGTACAGTTGACAGGTCAG**TTTTTTCTCCTTTCCC**
CCAAGCGGCCTGATGAAATCCTGAAAGAGGACA**TTTTTTCTCCTTTCCC**
GAATACGAAACCGGATAGCCAAGCCCTTTTTAAGAA**TTTTTTCTCCTTTCCC**
AAGCTTGAATCATGGT**TTTTTTCTCCTTTCCC**

Table S2. 6 linkers for a 60-helix bundle.

GTGAGGCGGTCA**TTTTTTCTCCTTTCCC**
GTTTACCAGCGC**TTTTTTCTCCTTTCCC**
CCATCGCCCACGCATA**TTTTTTCTCCTTTCCC**
GGGCCTCTTCGCTATT**TTTTTTCTCCTTTCCC**
GCGATAGCTTAGT**TTTTTCTCCTTTCCC**
CTATCATAACCCTCGT**TTTTTTCTCCTTTCCC**

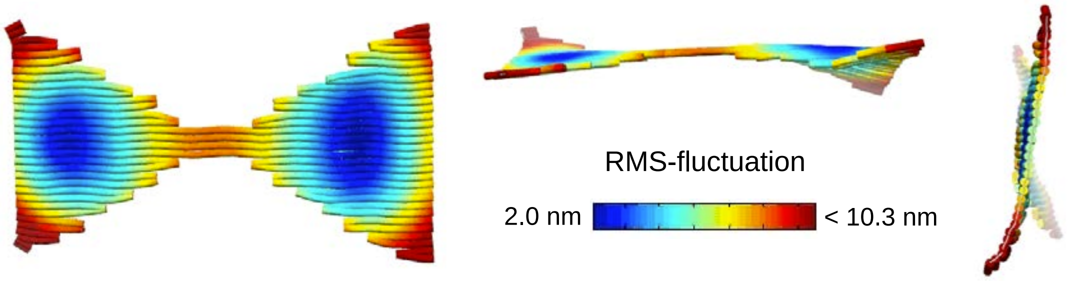

Figure S3. Predicted solution shape and fluctuations of a double-triangle. The simulation was performed using caDNAno design file as an input for CanDo modeling. Three views along different orthogonal directions are presented. The simulation based on a rigid-beam model of DNA indicates that a double-triangle is highly flexible.

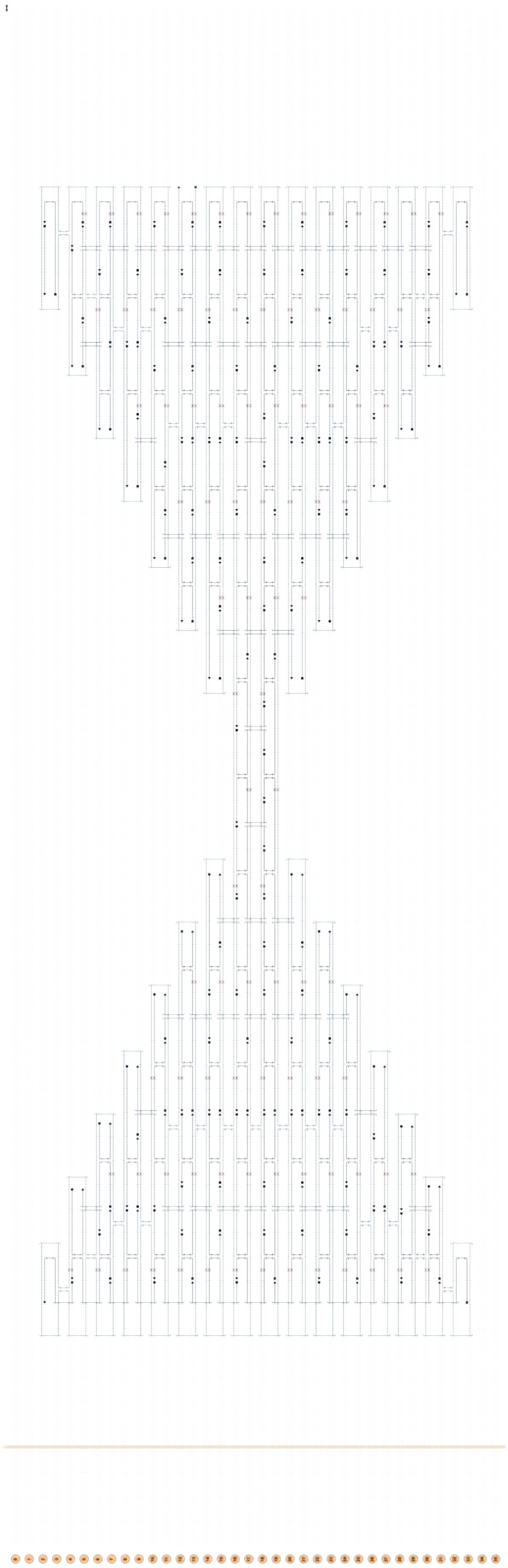

Figure S4. A caDNAno design for a double-triangle. Some of the bases of the scaffold strand have been skipped in the design in order to avoid global twisting of the shape.

Table S3. 163 staple strands for a double-triangle.

Start	End	Sequence
11[408]	10[392]	ACTATGGTGGGATTTTAGGGTCAGTTGGCAA
14[183]	11[183]	TAACTATATTCATCTTCTGACCTTAAATAAGGCGTTAAACAGTATAA
14[407]	11[407]	TATAAATCCACTATTAAAGAACGTGCGCTGGCAAGTGTAGGGCGCGT
22[87]	19[87]	CCCTCAGAGTCATAGCCCCCTTATAGCGTCAGACTGTAGTCACCAGT
4[431]	6[424]	AGAGATAGAACCCTTCAATATTACCGCCAGCCTGGTAATA
19[392]	22[392]	TTTTTTAAAATATTTTGTTAAAATAGAGAATCGATGAACGGGCTATCA
18[143]	19[143]	ATGTGAATATTGACGGGAAACCATCGAAGCGA
18[279]	16[272]	TCCAGCCAATCATGGTCATAGCTGGCAAAAGAAGATGAT
26[375]	24[368]	AATCCCCCGGTAATAGTAAAATGAATGCCTGAGTAAGAG
27[400]	26[400]	AGTCAGCATCAATTCTGCCTCAGAGCTACTG
25[392]	23[407]	GGATAGCGTCCAAATAAAGCTAAAAATTTTTAGAACCCTTGAGAAAG
7[400]	5[407]	AGGTGAGCAATACTTCGTCTTTAATGCGCGATTTTGAAT
27[352]	26[376]	CGAGAATGACCATAAATCAAAAATCAGGTCTATTCATTG
3[416]	2[440]	GTAATAAAAGGGACATTCTGGTTTTGACGCTCAATGGATT
19[296]	20[288]	GACAGATGCCAGAACGAGTAGTAA
17[144]	16[144]	AGGCGATTAAGTTGGGCTGTAAATCGTCGCT
18[199]	21[199]	GGGAAGGGTGATAAATTGTGTCGAACTACGAAGGCACCAAAGGACTAA
16[87]	13[87]	ATTTTGTCTGGCATGATTAAGACTCGAGGAAACGCAATAAATAATAA
15[120]	12[120]	ACGTAGACAGATAGCCGAACAAATAGCTATCTTACCGACTGAACAA
25[328]	24[344]	AATAGCGAGAGGCTTTTGCAAAAGCTCGTTT
21[288]	20[312]	AGATGGTTTAATTTCAACTTTAATCATTGTGGCCCTGAC
14[143]	15[143]	CAAAGAGAAAAGTAAGAAATACATACATTAAT
9[352]	11[359]	TCTAAAGCATCACCTTAAGGTTATCTAAAATAGAGGATTT
8[111]	9[111]	ATAGAATCTTACCAACTATTATTTATCAGCCA
18[247]	16[240]	CGGAAACCAGGATCCCCGGGTACAAACAAAATTAATTAC
10[87]	7[87]	GATTTTTTACAAAATAAACAGCCAGCTAACGAGCGTCTTTTGCACCC
27[424]	30[424]	TAGTAGCACATTTGGGGCGCGAGCTAGTTTGACCATTAGATGCGAACG
15[328]	18[328]	TTAACGTCCAAGTTACAAAATCGCTTCCACACAACATACGGTGCATCT
13[344]	11[343]	GCGGAATTCGTATTAAATCCTTTAATAGATA
6[103]	7[111]	TCCTTATCTTTTATCCTGATAAGT
21[424]	24[424]	TCATATGTAGAGGGTAGCTATTTTCAGTCAAATCACCATCTCAACGCA
27[112]	26[112]	CACAGACAGCCCTCATAAACAACTTTCAACA
15[424]	18[424]	GCGGTCCAGGGAGAGGCGGTTTGCGCTTTCCAGTCGGGAAGCGAGTAA
18[311]	15[311]	GACGACAGGTTATCCGCTCACAAGCAGAGGCGAATTATTAGAAATTG
22[391]	25[391]	GGTCATTGTAAAGATTCAAAAGGGCATATATTTTAAATGCTTTAGACT
18[327]	21[327]	GCCAGTTTAGGCGCATAGGCTGGCTCAGTGAATAAGGCTTAATTACCT
23[144]	22[144]	AATTTCTTAAACAGCTTTTGCGGGATCGTCA
24[367]	26[352]	CAACACTATCATAACCAAGTTTTGCCAGAGGGTCAAATGCTTTAAACA
7[440]	5[439]	CCTGAGTAAACTATCGGCCTTGCATTGCAAC
18[359]	18[376]	GTGTAGATTAATCTTGACAAGAACCGGATTTTTGTTAAAACCGTAAT
4[87]	2[80]	TTTTTCTACGGTATCATCGTAGGACGCCCAATAGCAAGCA
11[344]	10[328]	ATACATTTTCTTTAGGAGCACTAACAACTAAT
10[439]	7[439]	GAGCTAAAAAGTGTTTTTATAATACCGAGTAAAAGAGTCATCACTTG
4[118]	6[104]	CACTCATCGAGAACAAGCAAGCCATTCCAAGAACGGGTAGCTGTCTT
30[439]	27[439]	CCCAATTCTACATTTCGCAAATGTGTTTAGCTATATTTTTTAACATC
24[103]	24[120]	TTAAAGCCCTTTTGATGATAGCGGAGTGAGAATAGAAAGTATTCACA
30[103]	31[119]	ACCACCCTCGCCACCCTCAGAACCGCCACCCT
21[184]	18[184]	GGCTTTGCCTAAAACGAAAGAGGATTTGTATCATCGCCCGATCGGT
21[440]	18[440]	TGATAATCCAAAAACAGGAAGATCTGGCCTTCCTGTAGTCAACATT
18[119]	15[119]	CATTAAAGTTCAACCGATTGAGGACATATAAAAGAAACGGTTAGCAA
11[144]	9[159]	TTTAGTATCATATGCGGAATCGCCATATTTAGTAAAGTAATTCTGTC
16[215]	15[223]	CCTTTTTTGTGAATTTATCAAAAT
14[223]	15[199]	AGAGACTACCTTTTTAACCTCCGGCTTAGGTTCTGAGAAG

30[119]	28[111]	CGCCACCCAAGCCCAATAGGAACTACAAACTACAACGCCT
10[391]	13[391]	ATCAACAGTTAATGCGCCGCTACAGCGGTCACGCTGCGCGGTTTGGAA
12[103]	15[103]	AGCGCTAAAACAATGAAATAGCAAGTTACCAGAAGGAAACCCTTATTA
31[88]	28[88]	TTTAGTACGAGAAGGATTAGGATGGCTGAGACTCCTCAATCGGAAC
9[424]	12[424]	ATCCTGAGCAGGAGGCCGATTAAATGCTTTGACGAGCACGAGGAGCGG
28[139]	29[139]	TTTCGTCACCAGCCATGTACCGTA
17[296]	19[295]	GTGAAATTTATCGGCCTCAGGAAGTGAAAGAG
33[416]	33[439]	TATAATGCTGTAGCTCAACATGTT
18[423]	21[423]	CAACCCGTCAAAAATAATTCGCGTTGTATAAGCAAATATTCATGTCAA
6[423]	9[423]	TCCAGAACTTTGATTAGTAATAACTGTCCATCACGCAAATACGCCAGA
22[343]	19[343]	CTAACGGATTTAAGAACTGGCTCAAACAAAGCTGCTCATTGACCTTC
6[140]	7[140]	ACGAGCATGTAGATAATATCCCATCC
12[423]	15[423]	GCGCTAGGGGACTCCAACGTCAAAGTTCCGAAATCGGCAATGCAGCAA
18[215]	16[216]	AGGCTGCGGCCAAGCTTGCATGCCTGAATTA
24[423]	27[423]	AGGATAAATCGGTTGTACCAAAAAAAAATTAAGCAATAAAACTAATAG
9[400]	8[400]	TATCTACAGGAACGGTTAACCGTTGTAGGCG
23[368]	22[368]	ATAGTAATGTGTAGGCCTGAGAGTCTGAAGA
13[307]	12[307]	ATTATCATTTTGCTTTTAAAAGTTTG
13[328]	15[327]	GAAACCACTGGATTATACTTCTGATTTCAGGT
33[440]	32[416]	TTAAATATTACGGTGTCTGTTTGCGGATGGCTTAGAGCTT
18[263]	18[280]	GCACCGCTGTCAATCATAAGGGAACCGAACTGACCAACTTATCGCAC
21[200]	21[223]	AGACTTTTTCATGAGGAAGTTTCC
26[135]	27[159]	GATTTTGCTAGTTAGCGTAACGATCTAAAGTTTTGTCGTC
24[439]	21[439]	CCTTTATTAATATGATATTCAACGATAAATTAATGCCGGACCCCGGT
15[144]	14[144]	TTTCCCTTAGAATCCTAATCCAATCGCAAGA
15[440]	13[439]	TGCCCCAATCCTGTTTGATGGTGGGGCGAAA
21[328]	24[328]	TATGCGATACAACATTATTACAGGCCACATTCAACTAATGACGATAAA
20[367]	21[367]	TTACCCAAATCAACGTTTATACCAGTCAGGAC
23[168]	25[183]	ATAGTTGCAGCCTTTAATTGTATCTTCACGTTGAAAATCTCCAAAAAA
26[111]	27[111]	GTTTCACAGGAGTGTACAGTTAATGCCCATTC
21[368]	20[368]	GTTGGGGAGCAAACATCGCATTAAATATTCA
18[375]	16[368]	GGGATAGGCCTAATGAGTGAGCTCCAGTGAGACGGAAAC
20[407]	17[407]	TAAACGTTCCAATAGGAACGCCATCGGATTCTCCGTGGGGTTGCGCT
27[440]	24[440]	CAATAAAAAGGCAAAGAATTAGCCATTATGACCCTGTAGGGAGAAG
13[368]	12[368]	TTATCTGTTGTTCCATAACCACCACATTTAC
15[200]	18[200]	AGTCAATAAATGGAAACAGTACATTAAAACGACGGCCAGTCAACTGTT
10[183]	11[167]	CGCTCAACAGTAGGGCTTAATTGATTATACAA
30[423]	30[440]	AGTAGATTTCCTTTTGATAAGAGGTCATTGAAGTTTCATAGTTGATT
8[159]	9[135]	CAATAAACAACATGTTCAGCTAATGCAGAACGAAAGTACC
22[204]	23[204]	CCGATATATTCGGAACCATCGCCCAC
19[144]	18[144]	TTATACCAAGCGCGAAGCTGGCGAAAGGGGG
9[376]	8[352]	TCAAATACCTGCAACAGTGCCACGCTGAGAGCCAGCAGC
15[368]	14[368]	CGGGAGGCAACAGCTATAGGGTTGAGAGATG
5[440]	4[432]	AGGAAAAAAATACCTACACCAAC
14[167]	17[167]	CTGATGCATGAAAACATAGCGATAGTGAATAACCTTGCTTTAACGCCA
16[367]	18[360]	AATAACGGATTCGCCTTGTAAAGCCTGGGGTGTCACGTTG
11[168]	14[168]	ATTCTTACTAAGAATAAACACCGGATATATTTTAGTTAATTGTAAATG
16[183]	14[184]	ATATGTGAGCTTAGATTAAGACGGGGTTATA
15[288]	17[295]	CGTAAAACAGAAATAACATTTCAATTACCTGATTTCCTGT
9[112]	8[112]	GTAATAAGAGAATATCGCCTGTTTATCAACA
21[120]	18[120]	CAAAATCTAATCAGTAGCGACAGGGAAACGTCACCAATAAATTATT
19[344]	16[344]	ATCAAGAGGGGCGCATCGTAACCAGCCGGAAGCATAAAGGATTGCT
24[143]	26[136]	TTTCGATTGGCCTTGAGAACAACTAAAGGAATCTGTATGG
17[408]	14[408]	CACTGCCCGTATTGGGCGCCAGGCTGGCCCTGAGAGAGTAATCCCT
16[271]	18[264]	GAAACAAACATCAAGACGAGCTCGAATTCGTAGCTTTCCG
20[167]	23[167]	TACACTAAAACGAGGGTAGCAACGGGAGTTAAAGGCCGCTTGATACCG
12[367]	13[367]	AAACAATTCGACAACTATCATCATATTCCTGA

18[183]	16[184]	GCGGGCCTCCAGTCACGACGTTGAAATCAAT
16[343]	13[343]	TTGAATACAGATGAATATACAGTAATAATCCTGATTGTTCAGAAGGA
13[144]	12[144]	GAGAAAACTTTTTCAAAATCATAATTACTAG
2[439]	2[416]	ATTTACATTGGCAGATTCACCAGT
29[371]	28[371]	CGAAAGACTTCAAAAAAGATTAAGAG
16[391]	19[391]	TCTTTTCAAACTCACATTAATTGCAACAAACGGCGGATTGTCAGCTCA
12[204]	13[204]	ACCGACCGTGTGAAAATTTAATGGTT
24[183]	21[183]	CCAAAAGGGCCGACAATGACAACTCGCTGAGGCTTGCAGGCTACAGA
12[143]	13[143]	AAAAAAACTGAACACCAGCCCTTTTTAAACGC
7[371]	6[371]	GAACGAACCACCACATCGCCATTAAA
22[143]	23[143]	CCCTCAGCCACCACCGCAGGTCAGACGAGGTG
15[104]	18[104]	CGCAGTATCAAAGACACCACGGAAAGACAAAAGGGCGACAGTGAATTA
30[407]	28[400]	GGAAGCAACGAGCTTCAAAGCGAATGAAAAGGTGGAAGC
23[307]	22[307]	AGATTTAGGAATATAGAAAGATTCAT
8[399]	9[399]	GTCAGTATTAACACCGTCAAACCCTCAATCAA
31[392]	30[408]	GGATTAGAGAGTACCTTTAATTGCCCAGACC
28[399]	30[392]	AAAGCGGATTGCATCAATATCGCGTTTTAATTACTCCAAC
7[112]	5[118]	CCTGAACAAGAAAAAAACCAATCAATAATCGTTAAACC
16[239]	18[232]	ATTTAACAATTTCATTTGCAGGTCGACTCTAGAGGCAAAG
25[88]	22[88]	ATACATGGAGAATGGAAAGCGCACCAGAACCACCACCAGACCGCCA
22[367]	23[367]	AAAATCTACGTTAATAAAAGGAATTACGAGGC
15[312]	14[288]	CGTAGATATAATGGAAGGGTTAGAACCTACCATATCAAA
21[104]	24[104]	GCCATCTTAGCCGCCACCCTCAGAAGCCGCCGCCAGCATTAATCCTCA
24[343]	22[344]	ACCAGACGCAGATACATAACGCCAAAACGAA
33[80]	31[87]	AAGTATAGCCCGGAATCCGTACTCAGGGATAAAGGCGAGG
17[168]	20[168]	GGGTTTTCCTTCGCTATTACGCCAACAAAGTACAACGGAGCAAAAGAA
10[111]	12[104]	TTTCGCCAATCCAAATAGAATAACATAAAAACGGTAATTG
18[231]	18[248]	CGCCATTCCATGTTACTTAGCCGGAACGAGGCGCAGACGTCTGGTGC
13[88]	10[88]	GAGCAAGATATCAGAGAGATAACAGCAGCCTTTACAGAGAAGAAAC
13[392]	16[392]	CAAGAGTCAAAAGAATAGCCCGAGGATTGCCCTTCACCGCGTGGTTTT
20[143]	21[143]	CCCCCTAGCAGCACCGACCGGAACCAGAGCAG
24[119]	21[119]	AACAAATAGACAGGAGGTTGAGGGAACCGCCTCCCTCAGTTCATAAT
28[87]	25[87]	CTATTATTACAGTGCCCGTATAAACTGGTAATAAGTTTTTAAGCGTC
12[119]	10[112]	AGTCAGAGAGGGAAGCGCATTAGATTTAGGCAGAGGCAT
7[88]	4[88]	AGCTACAAGAGGTTTTGAAGCCTACCTCCCGACTTGCGGGTTTTTA
23[408]	20[408]	GCCGGAGATGAGAGATCTACAAAGTAATCGTAAAACTAGTAAATTG
9[136]	11[143]	GACAAAAGACAACGCCAACATGTAACGGGAGAATTGCCTG
20[311]	18[312]	GAGAAACAAACGGTGTACAGACCGAGGGGAC
16[143]	17[143]	ATTAATAAAGGTGGCAGAGGGAAGGTACTGCA
18[439]	15[439]	AAATGTGAACCTGTCGTGCCAGCAATCGGCCAACGCGCGCGCTGGTT
20[223]	18[216]	TAAAATACGTAATGCCAATCCGCGACCTGCTCGCCATTC
5[408]	4[392]	GGCTATTATGACCTGAAAGCGTAAGAATACGT
28[110]	30[104]	GTAGCCCTGCCTATTCATTTTCAGGGATAGCTCAGAGCC
19[88]	16[88]	AGCACCATACCGACTTGAGCCATGGTTTACCAGCGCCAATAAGTTT
18[103]	21[103]	TCACCGTCTACCATTAGCAAGGCCAATCAAGTTTGCCTTTTAGCGTTT
11[328]	13[327]	GAGCCGTCGCCCGAACGTTATTAAGGAACAAA
13[440]	10[440]	AACCGTCAAGGGAAGAAAGCGAATATAACGTGCTTTCCCAGAGCGG
26[399]	27[399]	CGGAATCGTCATAAATTTACCCTGACTATTAT
5[392]	7[399]	GACAATATACTGATAGCCCTAAAAGCAGAAGATAAAACAG
11[360]	9[375]	AGAAGTATTAGACCCCGCCGCGCTTGAAAGGAATTGAGGGCTGAACC
14[367]	15[367]	ATGGCAATTCATCAATACAGTACCTTTTACAT
21[144]	20[144]	CGAAAGACAGCATCGGAACACTCATCTTTGA
26[159]	24[144]	TTAGTAAATGAATTTTTGCGAATAATAATTTTGGTTTATCAGCTTGC

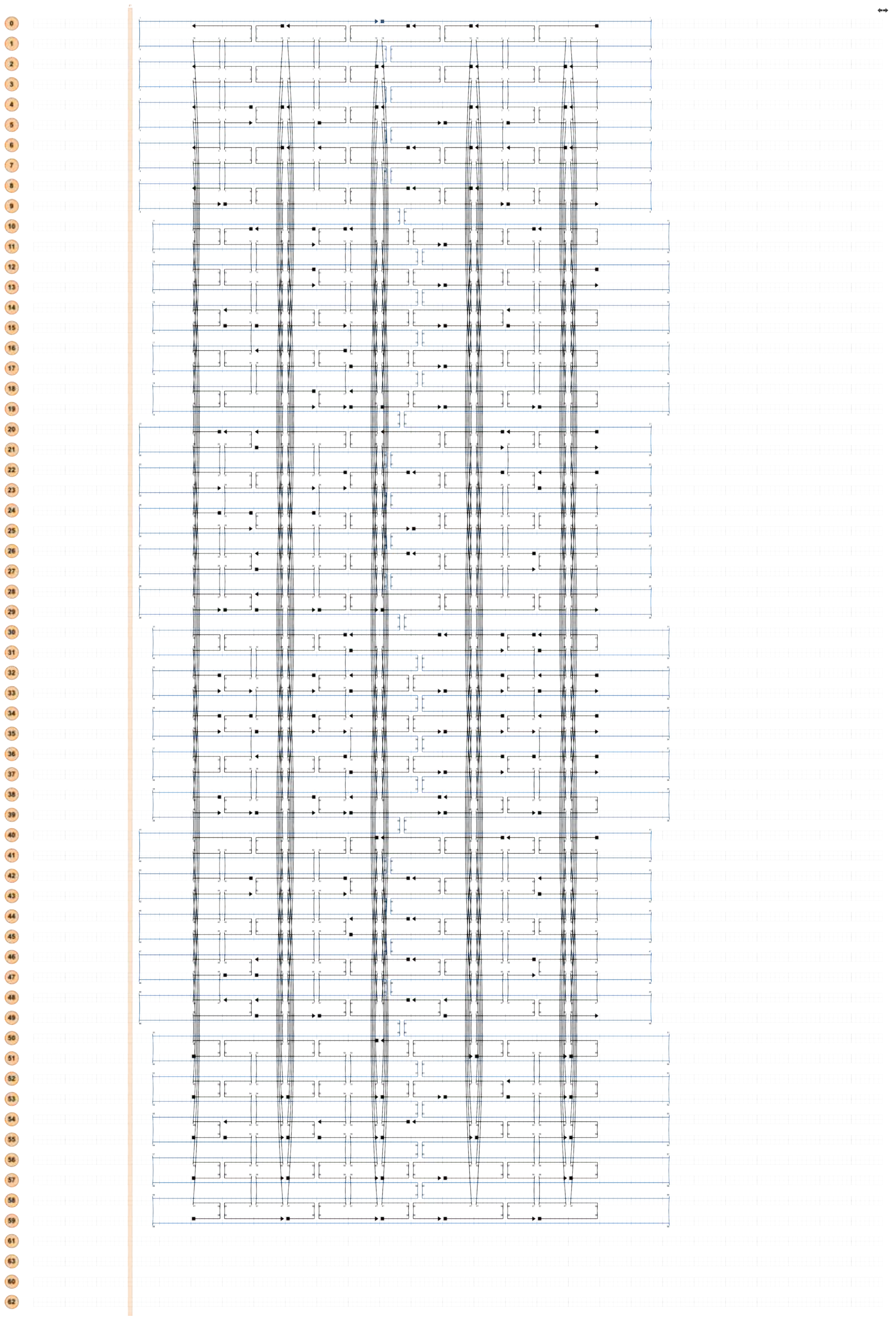

Figure S5. A caDNAno design for a 60-helix bundle.

Table S4. 141 staple strands for a 60-helix bundle.

Start	End	Sequence
38[90]	20[77]	GATACTTGCCAGTTAAACCATCAATATGGAGCAAACAACAAA
36[69]	39[69]	GGAGGTTCGAGCGTAAACAGCGAGAGAA
42[83]	22[84]	CTTGCAGGAGCCTACCTCCCGGACCTTTATTTCAAGACAGTC
39[112]	57[118]	AAACGAATTATGCGGGGCTTGCAGAACCACCCATG
39[91]	59[90]	GAAGAAACTCATTACACCAGACACCACCCTCATCTGGTTGAT
53[105]	59[111]	ATTTTTTCGGAGTGTCTTTCCCACAGACAATAGGAGCCACCCGAATAGG
17[91]	37[90]	ATATTTTGGCCGGACGCAAGGAAAGAAGTTTTGAAATATAAC
32[41]	9[41]	AGCATGTAAGAATAGCCAACGCAGTTGAAATATCTCCTGCAA
30[69]	35[69]	TAAGAGAAACGCGCGAAAAATGGGTATTAGCCGTTTAAGAAC
26[83]	6[84]	CATCCTATTTTAAAACTAATAGCTCCTGTGTGAAAGCGTTGC
4[118]	32[112]	CGTATTGCAGCTGCGGTCACGCGACAGTGGTGGCAAATAACCAGGAAGC
15[42]	20[49]	AGATGATAAAATTATAGATTTTTGCTTTATTATTCATTTCAATACATTT
24[41]	6[35]	ACTATATGATTATCGGAGCGGCAGATTCGAATACG
29[77]	10[70]	CATTTATTGCTGAAACGAGTAGATTTGTGAGCACTTTAGCATCACCTTG
32[62]	11[62]	AATATCCAATTACTACAAATTGGTTATC
57[119]	35[125]	TACCGTACGCCTGTGAGTAATTCATAAGATAGCGT
33[112]	51[118]	TTACCCTACCAAGCCAAAAGAGTTTATCGACAATG
26[111]	20[105]	TCAATTCATTAGCACATTATGTTTTTAGAAAAGGGTAGCTGAAGGTCAT
36[111]	39[111]	GAGAGGCCGAGGCAATACCACGTTAATA
53[119]	29[125]	AAAATCTTGTATCGATACACTACTTTTTAAACTCCCTCCTTTATATGCA
32[104]	9[104]	ACTTCAATTTCGCATCCCAATCGCAACTTTAAGTTTGTAAAA
11[91]	30[91]	CAGGCTGTCTGCGATATAATGGATGGCT
46[111]	40[105]	GCGAAACGACCTGCAACTGACCTGACCTAACAAAGGTAAATTATTTTAA
4[48]	0[35]	TTGACGCTAATATCCATCACTCCGAGTAGGTACGCCAGAATC
15[91]	35[90]	ACATTAAAATACTTAGGCAAGCAGAAAACGAGATCCGCCAGA
16[69]	19[69]	AGGGTTAAGATGAAAACAATAAGCAAAA
12[125]	10[112]	CACCGCTATACGAGGATCCCCGGGTACCCAGTGCCGTGCTGC
9[42]	5[48]	CAGTGCCAAGATAAGTCTTTAAGCGTAAACCAGTC
33[91]	53[90]	CATAAATAACGGAGACCAACCGTGAATTTCTTAGCAAAAAGG
2[76]	34[70]	GTAGCAACATTGCATAATGGATGATTGTACCTTTTTATTTTAACAAGCA
49[91]	51[97]	AGCGAAACTTTTGCGGGATCGTTAACAGCTTGATA
31[70]	48[84]	CTGTTTAATTATTCAAACGTCACCAAACCCTCAGCAATACGT
2[118]	34[112]	AGGCGAACCCTGAGTAATTCGCAACCCGCCAAAAAAAATTAACCTCAAA
17[70]	44[70]	TATACAGGTCGCTATCTGAGAACTTGCGGCGAGGCAATACCC
35[49]	53[55]	CTTATCCGATTAAGGGCAACAGGAGGTTACCCTCA
6[83]	29[76]	GCTCACCAACAGAGAAACATCGATTAGATCTTTAGATCATATGCAGAGG
57[35]	35[41]	GGGTCAGAGTGTACCGAACAAATTACGCTCAGATA
6[97]	13[90]	ATTAATTTTGTTATAAGCGCCATTCGCAAGGTGCA
53[77]	46[84]	CCACCAGCATCTTTGGCACTACGAAGGCATTTGTA
19[70]	38[70]	GAAGATGCATCAAGTTCTGTAACAAAAT
40[76]	57[76]	AGCGCATAATAATAAACCTATAGTTAAT
45[70]	53[76]	AAAGACAGAGCCGCAGAGCCG
38[62]	4[56]	CATATTAGTGAATAATTTTCCTAACGTCGAACCTAATATTACGAAATAC
30[104]	27[111]	TTTTGCGCTGTAGCTCAACATAGTTGATAATGGTC
4[97]	4[77]	TTTTCTTTTCACCAGTGAGAG
33[70]	50[77]	AAACCAAGGAATAATATTGACTCATAATCCCCTTATTAGCGTTCTGAAA
51[119]	49[125]	ACAACAAGCTGAGGGCAACGG
29[42]	25[48]	TATTTAAGTATAAAAACACCGTTTGAAAAAAGAAC
4[76]	32[70]	GAAAAACACATTCTCATTATCTTATTAAATTTCATGCCTGTTTGAACAA
44[83]	25[83]	GAACGGGGTTTTAGATCGAGAGTAATAA
22[125]	4[119]	GTAGGTATTGTTAATCAAAAAAGAGTTGCGGTTTG
57[91]	35[104]	GGGATAGCAAGCCCAGCCCTCAGGCTGGCAACTTTATAGTAA
18[62]	16[49]	ACGGATTCTGTCCATTGATTAGTAATAACAGAACACCATATC

42[69]	39[62]	CAATGAATAAGCCCTAGACGGGAGAATTCTTTACA
22[83]	2[77]	AAATCTCTAACAGTTTCTGAAACACGGGCAACAGCCGAAATCGGCAATT
10[48]	14[42]	CTCAATCAAGGAATTGAGGATAACTCGTACCAGAA
55[63]	59[76]	AATGGAACGTCATATATAAACTATTCTGGCGGATAAGTGCCG
0[125]	22[112]	TATTAAAGAACGTGAGGGTTGGAAGATTTAAATTTAAGATTC
0[83]	18[70]	TCTATCAGCCGATTAATTAACCGGGAGA
48[83]	48[91]	AATGCAATCAACAATAAATTCGAGCTTCCGGGTAA
49[63]	43[69]	AGGCCGGATTAAAGAGGTAAAGTTTATTGAAACGCAAAAGAAGAAACGC
22[69]	19[62]	TTAATTAACCTTGCAAAACAAAATTAATTTACCTG
35[91]	54[84]	GGGGGTAGAAAGAGGTGTCGAAAAGGAACAACTCC
38[41]	4[35]	AATAAGATACATAAAAACATAAATTGCGTTTGCACTTGCTGGTCAATCG
6[55]	28[49]	ACCTGAAATGCGCGATACATTTGAGGAACTTACCA
21[49]	55[55]	TATGTGATTTATCCCTTACCATAGCTATAGAAGGATTTGATGCTCATTA
46[83]	26[84]	TCATCACGTACCGCTAAGTCCTAAGTTTGACCATTGCATTAA
12[62]	10[49]	ATAGATAAACTGATCGAACCACCAGCAGACGCTGATCAAACC
34[48]	39[48]	AGGAATCTAGAAGGCAAGATTCCTGAATCAATCCAATGAAAA
30[111]	35[111]	AGGTCATACCAGACCCGAAAGCAGGTCTTGCTTTAAATGTTT
34[62]	13[62]	TTTATTTAACTTTTCCTAAATCCTTTGC
29[49]	33[48]	CAACGCCGTACCGACAAAAGGCAACATGATTTACGTTTCCTT
43[112]	55[118]	TCATCAAAGCATTCAGACGTT
53[56]	29[62]	GAGCCACACCGGAAGGAGGGAGTGAATTAATGCAGATATAAAAACATGT
39[49]	57[55]	TAGCAGCAACTGAACACAAGAAAAGTATAGTAACA
49[49]	54[42]	CATTACCTCAGTAGCGACAGACGTTTTCCACCACCAACCGCCGAGGCAG
29[63]	23[69]	AATTTAGGCGTTATAGAAAAACTTCTGATCAAATATAACCTCTCATAGG
24[62]	6[56]	CGGCTTACATCAATGGAACAACAGTAATCCTTCTG
5[91]	15[90]	CAGTCGGCGCATCGTAACCTTATCATCA
9[105]	6[105]	CGACGGCGAGCTCGCAATTCCGTGAGCT
8[97]	11[90]	AATCATGACGACGTGGGTAACGCCAGGGAACCATT
5[105]	0[98]	GTCGTGCGGCGCCACGCCTGGAATCCTGCCGAGATGACTCCAACGTCAA
50[76]	54[63]	CCATCGATAGCAGCTCATAGCCAAAATCCACCCTCCGCCAGC
10[69]	15[69]	CTGAACCTAAAATAGCCGTCACCGAACGATTTTGCATAATCC
30[90]	30[70]	TAGAGCTTATCGAGCCAGTAA
10[111]	14[105]	AAGGCGAGTTGGGACCGGAAAGGGACGATTGGTGT
15[105]	19[111]	CGAGTAACGTCTGGTTCGCATGTATAAGTAAAACT
20[41]	2[35]	TCATTTGGAGGCGAGAATACCGAGGCCATGCCTGA
47[49]	49[62]	GATTGAGCCAGAGCATCGGCATTTTCGGACCGTAAATTAGCA
57[77]	42[84]	GCCCCCTACCGTTCACGTGTACAGACCAAATAAGG
51[98]	47[111]	CCGATAGAAGGCCGGACAGCATCGGAACAAGTTTCAAAGAGG
34[41]	8[35]	ATTACCGGCAAGACTACCGACATTCGACTTAGAAGGCTATTAAACAGAG
32[125]	9[125]	GATTAAGTGTTTAGTCATTCCTCGGTGCGGGGGATAAGCTTG
59[56]	23[62]	GTACCAGAAACATGATTGAGTATAGCAAACGCTAATTGAAGCATCAAAA
15[49]	46[49]	GGCAATTGGTTGGGGCGAGAATCATCGTATCATTCAATCAAT
0[55]	36[49]	CAGGAACAAAGAGTCGCCTGATCAGGTTCTTAGAATGAATTTCTTAAAT
6[118]	30[112]	CTAATGAACACAACTCTGGTGAGGGCGAATATAACGTTTTAATGATAAG
40[104]	21[104]	GAACTGGAATCTACATTCAACACCGTTC
55[35]	33[41]	CAAACAAGTCAGACACATACATCATATGGGCTGTC
8[83]	8[84]	CTGTTCATTAAAAATGAAAAATCTAATTCCCAGTCGTCATAG
40[125]	21[125]	AATTACCCTAACGGGTTGAGAATGCCGG
55[77]	55[97]	TCTCTGAATACGATCTAAAGT
55[98]	33[104]	TTTGTCGAGAATAGAATCCGCAAAGTACCAAAAAT
36[125]	6[119]	AAAACCAATATATTGGTTGTATCGGATTTGGGATAATTAATGGGGGTGC
59[91]	37[104]	ATAAGTATAGCCCGTCAGAGCACGAGTACTGCTCAGGAATTA
54[83]	44[84]	ACCACCACCGCCTGATAAATTGACAGAT
59[77]	40[77]	TCGAGAGATTTCGGAGCCTGACGAGAAATACCAGTCAAGGGA
4[55]	26[49]	CTACATTACACGACAGAAACCATTAAATTTAATGG
42[48]	39[41]	CTTACCGGATAACCCACCCTGAACAAAGGTCAAAA
5[63]	0[56]	AAAAGGGGCTCATGCGCCAGCTACTTCTTCACGCAAAAGGGATTTTAGA

25[84]	5[90]	ATCATACTTGCGGGAGAAGCTTTGGATTTGAGTAAGGCTGCCCGCTTTC
31[112]	52[105]	CGGAAGCCATGAGGGAGGGTACTTGCAGGGAGTTATTGCGCCAGCTTGC
53[35]	29[41]	CCCTCAGGGAACCGGGCGACACTTGAGCCAATAAATAAAGTAATCGCCA
2[97]	17[90]	GTGGTTCTGATTGCGTAGCCAGCTTTACACCGTTA
20[104]	2[98]	TGCCTGAGTAATCGCAAATATGAATAGCTTTGATG
10[62]	6[63]	TCAAATAGAGCCAGCAGCAAATACCGAAAGCCCTAATAGAAC
36[104]	6[98]	TTTTGCAATAAAAAACCCTGTATGTGAGAGATGGGGAAACCTAACTCAC
55[42]	59[55]	ATAAATCATACAGGTGCCTTGTAAGAGGCGGGGTTTTGCTCA
37[70]	55[76]	CTTTCCACAAGAAAAATAATACAGTAAGAGCGCAG
34[104]	8[98]	AACAGTTGCAAAGATACTAATGTTTGAGCCAGGCACCGCTCAAATTCGT
20[125]	2[119]	CTACAAACAATCATAAAACAGAGTGTTGCCCCAGC
24[48]	20[42]	TTATATATCAATAGTCCTTGAATCAATAAACAATT
53[91]	31[104]	AATTGCGAATAATATTTCGAGTAAAACGCATTAAAAAAGCGA
19[91]	38[91]	ATGAACGGAGTCTGATATTCATAATGCA
0[97]	19[90]	AGGGCGAATCAAAATTAAATTGTAAACTAGAATCG
37[91]	57[90]	GCCAAAATTCAGTGGGCGCATATAGTTAGCGTATTGCTTTCA
55[56]	33[62]	AAGCCAGATTGACATATAAAATTGTCACCAAGAAC
59[35]	23[41]	GGATTAGCTGAGACTCAGAGAAAGCCCTATTTTATAGTTGCTAGAAGAG
59[112]	37[125]	TGTATCACCGTACTCCACCCTAGATGGTACCCAAAAGCAACA
39[70]	39[90]	TAACATAAAAACGGACGTTGG
23[112]	53[118]	AACCCTCAAATAGCAGACTGGGGAACCGTCCATGTGTTTCAGCACGTTG
51[35]	48[42]	TGTAGCGATCAAGTATCACCAGTAGCACTCACCGA
55[119]	33[125]	AGTAAATTTCAACATACTTAGCGATTATGACTATT
47[42]	43[48]	TTCAACCAGAAAATTAAAGGTACTCCTTAGTTACC
22[104]	4[98]	TGAGAAAGTTAAAACCTTCCTCCTTCACGGGTGGT
27[49]	48[49]	GAATCATCATCCTATTCAGCTATCACCG
34[125]	13[125]	GAATCCCGCAATAACTGAAAAATCGGCC
19[112]	42[112]	AGCATGTGGCTATCTAAATTATTTAGGATAGTAAGTCAACGT
19[77]	0[84]	ATGAAAGTTTACATCGAATCCCTTATAAAAAACCG
13[91]	33[90]	TCTGCCAAGTAGTAAGATACAATATCGCGTTTTGAACATGAC
57[56]	35[62]	GTGCCCGCATGGCTAACCGAGCTGGCATGGTATTC

Supplementary information references

[S1] W. Liu, H. Zhong, R. Wang and N. C. Seeman, *Angew. Chem. Int. Ed.*, 2011, **50**, 264-267.

[S2] V. Linko, M. Eerikäinen and M. A. Kostiainen, *Chem. Commun.*, 2015, **51**, 5351-5354.
